# Trends and Factors Affecting Hospitalization Costs in Patients with Inflammatory Bowel Disease: A Two-Center Study over the Past Decade

**DOI:** 10.1155/2013/267630

**Published:** 2013-11-07

**Authors:** Junjie Xu, Minyue Tang, Jun Shen

**Affiliations:** ^1^Department of Medicine, Renji Hospital, Shanghai Jiao-Tong University School of Medicine, 160 Pu Jian Avenue, Shanghai 200127, China; ^2^Department of Medicine, Ruijin Hospital, Shanghai Jiao-Tong University School of Medicine, 197 Ruijiner Road, Shanghai 200025, China; ^3^Department of Gastroenterology, Renji Hospital, Shanghai Jiao-Tong University School of Medicine, Shanghai Institute of Digestive Disease, Shanghai Inflammatory Bowel Disease Research Center, 160 Pu Jian Avenue, Shanghai 200127, China

## Abstract

With the growing number of patients with inflammatory bowel disease (IBD) and hospitalization cases, the overall medical care cost elevates significantly in consequence. A total of 2458 hospitalizations, involving 1401 patients with IBD, were included from two large medical centers. Hospitalization costs and factors impacting cost changes were determined. Patients with IBD and frequency of hospitalizations increased significantly from 2003 to 2011 (*P* < 0.001). The annual hospitalization cost per patient, cost per hospitalization, and daily cost during hospitalization increased significantly in the past decade (all *P* < 0.001). However, length of stay decreased significantly (*P* < 0.001). Infliximab was the most significant factor associated with higher hospitalization cost (OR = 44380.09, *P* < 0.001). Length of stay (OR = 1.29, *P* < 0.001), no medical insurance (OR = 1.31, *P* = 0.017), CD (OR = 3.55, *P* < 0.001), inflammatory bowel disease unclassified (IBDU) (OR = 4.30, *P* < 0.0001), poor prognosis (OR = 6.78, *P* < 0.001), surgery (OR = 3.16, *P* < 0.001), and endoscopy (OR = 2.44, *P* < 0.001) were found to be predictors of higher hospitalization costs. Patients with IBD and frequency of hospitalizations increased over the past decade. CD patients displayed a special one peak for age at diagnosis, which was different from UC patients. The increased hospitalization costs of IBD patients may be associated with infliximab, length of stay, medical insurance, subtypes of IBD, prognosis, surgery, and endoscopy.

## 1. Introduction

Inflammatory bowel disease (IBD) is an immune-mediated, chronic, idiopathic, and relapsing inflammatory condition of the gastrointestinal tract, consisting of three subtypes as Crohn's disease (CD), ulcerative colitis (UC), and inflammatory bowel disease unclassified (IBDU)  [1, 2]. Over the past decades, the incidence and prevalence of IBD were increased across the globe and vary in different geographic areas [3, 4]. Originally, IBD was more common in developed, industrialized countries and regions (such as Northern and Western Europe as well as North America), implicating urbanization as a potential risk factor. However, in recent years, trends in epidemiological data suggest that the incidence distribution of IBD is changing [5]. Recent reports indicate that regions or countries, such as Eastern Europe and Asian countries, with previously low-incidence, are now experiencing a continuous rise in incidence [4, 5], whereas the incidence in most Western European countries and North America has stabilized or slightly increased, with decreasing rates reported for ulcerative colitis [6, 7]. Another parallel evolution was a trend for the previously reported predominance of UC to give way to CD in developed nations, as CD became more prevalent. However, in some well-developed nations (e.g., Japan) and developing countries (e.g., China and Eastern European countries) where IBD is emerging, UC still predominates [6]. Over the past years in China, the incidence of IBD has been progressively increasing and the proportion of its subtypes has also changed. It was suggested that the rate of hospitalization cases increased fivefold in the last 5 years compared with the first 5 years by a retrospective nationwide survey in the years 1990–2003 [8]. Furthermore, the peak prevalence of UC and CD occurs in the 40- to 49-year old and 20- to 50-year old brackets, respectively, and no bimodal age distribution was observed. There is still a lack of data and reports available on the recent epidemiology of IBD which can provide a clue indicating IBD epidemiologically differs in China from Western countries.

With the increasing number of IBD patients and hospitalization cases, the overall medical care cost elevated significantly in consequence, laying substantial burden on healthcare resources [9, 10]. However, a cost analysis of health care in a European IBD inception cohort with 10 years of follow-up evaluation found that the mean total cost of IBD care decreased sharply in the second year compared with the first year and fluctuated little thereafter, which was probably related to the natural progression of the illness as modified by different therapies [11]. Among the overall healthcare expenditure, medical and surgical hospitalizations were reported as the most expensive resources in Europe [11, 12]. Recently, since the advent of effective but relatively expensive biological agents, the contribution of surgery costs has gradually given way to medication costs [13, 14].

Nowadays, it is an era of escalating healthcare costs and increasing constraints on healthcare budgets. In China, the increasing health-care burden on the national medical health system as well as the society driven by elevating prevalence of IBD attracts great attention from gastroenterologists and administrators over the country. Thus, understanding the trend, the proportions and predictive factors for the cost of IBD are critical and urgently required for cost-effectiveness evaluation, treatment modalities, determination of health insurance policy, and proper distribution of healthcare resources [9]. Economic analyses in the US and European countries may not apply to the situation of developing countries due to the different patterns of health care and payment systems between countries.

In the present study, we aimed to analyze the composition of the hospitalization cost on the basis of medication, endoscopy, and surgery, and to figure out its alteration and predictors over the past decade. In addition, we also explored the demographic and epidemiological characteristics changing with era and tried to discover a pattern of epidemiological condition in patients with IBD.

## 2. Patients and Methods

### 2.1. Data Collection

This study was approved by Research Ethics Committee of Shanghai Jiao-Tong University, School of Medicine. Data includes patients with inflammatory bowel disease hospitalized at Renji hospital, Shanghai Jiao-Tong University, School of Medicine, from Jan 2003 to May 2012 and Ruijin hospital, Shanghai Jiao-Tong University, School of Medicine, from Aug 2009 to June 2012 with a diagnosis of “Crohn's disease (ICD10-K50),” “ulcerative colitis (ICD10-K51),” or “inflammatory bowel disease unclassified (IBDU).” Data was analyzed retrospectively using a computerized database and double checked according to ICD numbers. Demographic and clinical information (subtype of disease, name, age, gender, contact number, address, insurance status, check-in and check-out date, prognosis, urgency on admission, surgical information, endoscopic information, and infliximab use) was recorded in a prepared table.

### 2.2. Demographic and Epidemiologic Analysis

Demographics, such as name, age, gender, contact number, and address, were used to recognize hospitalizations of the same patient. By excluding the reduplicative hospitalizations of the same patients, the number of patients was determined. Population data of IBD patients and hospitalizations were collected from Renji hospital between Jan 2003 and Dec 2011 and from Ruijin hospital between Jan 2010 to Dec 2011. The mean ages at diagnosis in subgroups of IBD patients were calculated according to the medical records. Age data was collected according to the check-out date of the first hospitalization of a patient. The insurance status was categorized as “medical insurance” or “no medical insurance.” The term “medical insurance” referred only to public medical insurances provided by the government. And the term “no medical insurance” included expense paid by patients themselves or insurance companies with different reimbursement rates. The hospitalization year was recorded according to the check-out date.

### 2.3. Determination of Total Hospitalization Costs, Proportion, Alteration, and Predictors

Data of hospitalization costs was collected from the computerized database of each hospital, including medications, laboratory tests, examinations, ward bed, surgery, nursing, treatment, and consultation. The cost of biological agent (infliximab) was included in medication. The cost of endoscopy was included in examination. Surgery cost included surgical consumables, anesthetic fee, and operation fee. Hospitalizations were categorized as either “surgical” if a primary IBD-related surgery was performed during this hospital stay, or “medical” if either no surgery or a surgery unrelated to IBD was performed. The cost was averaged by the number of hospitalizations, patients, and length of stay (LOS) [15]. “Annual hospitalization cost per patient” was defined as the annual hospitalization costs averaged by annual number of individual patients, reflecting the real burden for every IBD patient. “Cost per hospitalization” was defined as hospitalization costs averaged by the number of hospitalizations during a certain period. “Daily cost during hospitalization” was defined as hospitalization costs averaged by LOS during a certain period. “Cost per hospitalization” and “daily cost during hospitalization” can show more patterns about the changes and factors controlling these changes.

Several individual variables included in our study were addressed as follows. First, infliximab (IFX), the only biological agent used in IBD patients in China, an antitumor necrosis factor (anti-TNF) chimeric monoclonal antibody, was used in these two medical centers since 2008. Thus, we included IFX data from 2008. Second, there were mainly three types of endoscopic examinations: gastroscopy, enteroscopy, and colonoscopy. The main type of enteroscopy was double balloon enteroscopy, while only a few patients experienced push enteroscopy. Endoscopic data in Renji Hospital from Apr 2010 to Mar 2012 were collected. Thus, we calculated the endoscopy proportion of both examination and total hospitalization cost. Third, operations included incision of perianal abscess, anal fistula fix, intestinal anastomosis, total intra-abdominal colectomy, ileostomy, intestine exteriorization, transverse colostomy, sigmoidectomy, Hartmann's rectum resection, enterolysis, and exploratory laparotomy.

To analyze factors impacting the hospitalization cost and the changes, average hospitalization cost was classified as an ordinal variable by lower quartile, median and upper quartile. Ages, genders, LOS, insurance status, disease subtypes, prognosis, urgency on admission, surgical intervention, IFX, and endoscopy entered into the ordinal logistic regression model [16]. The prognosis was categorized as “died or not improved” or “cured or improved.” The urgency on admission was categorized as “critical or urgent” if patients were admitted in emergency room or “normal.” The categorical variable of “disease subtypes” was transformed into dummy variables in order to fit in the logistic regression. Odds ratios (OR) and their 95% confidence intervals (CIs) were calculated for binomial variables.

### 2.4. Statistical Analysis

Descriptive statistics were used to characterize the study population. Continuous variables are presented as the means and 95% CIs, and categorical variables were presented as numbers and percentages. Regressions, including linear, exponential, and logarithmic, were applied to explore and demonstrate the tendency. We compared *R* ^2^ values and *P* values of the regression models in order to choose the better ones as appropriate for estimating and reflecting the tendency. All the *P* values were calculated for two-tailed, and 0.05 or less were considered statistically significant. Analysis of variance was used to compare the average cost among different groups. Chi-square test or continuous correction chi-square test or Fisher's exact test was used to compare the proportions among different groups. Furthermore, eta-squared, the ratio of unique sums-of-squares to total sums-of-squares was used in the present study as well [17]. PASW Statistic 18.0 and Microsoft Office Excel 2007 were used for statistics, tabulation, and plotting.

## 3. Results

### 3.1. Increase of Hospitalization and Different Patterns in Subgroups of IBD

A total of 2458 hospitalizations, involving 668 CD patients, 665 UC patients, and 68 IBDU patients were enrolled in our analysis. Males represented 63.38% of all IBD hospitalizations, 61.03% of all IBD patients.

**Figure 1:**
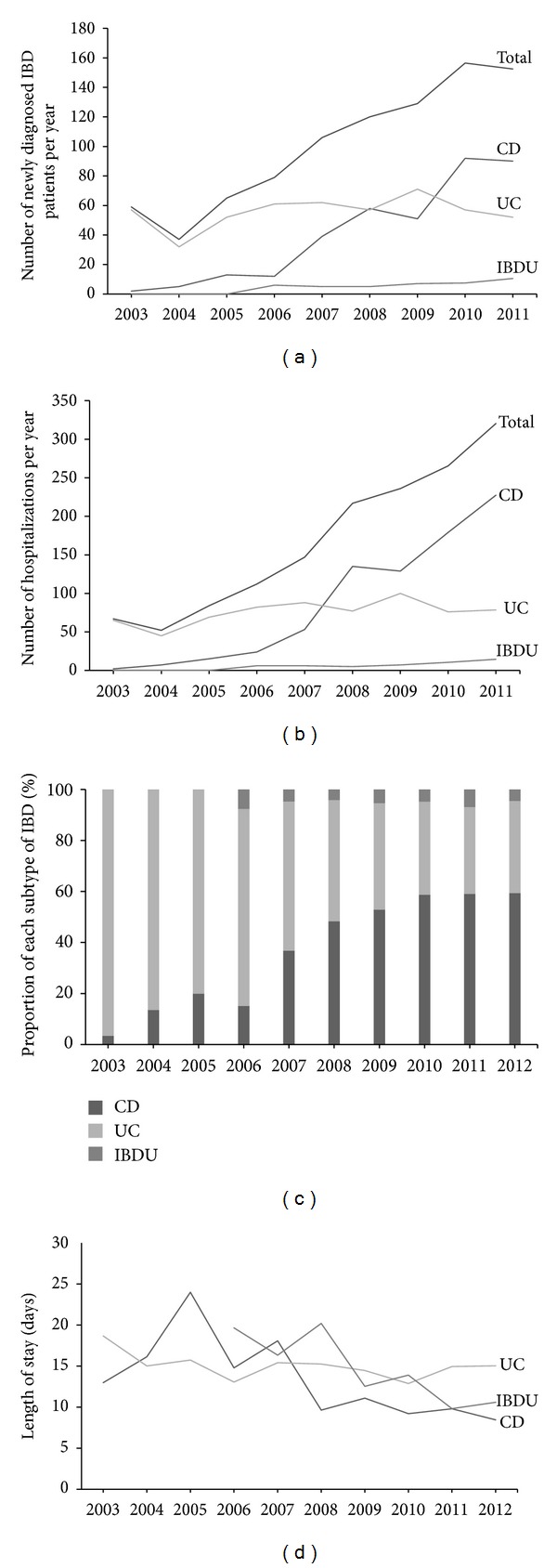
Growing population of IBD patients and hospitalizations. (a) Number of newly diagnosed patients with IBD per year. CD patients increased more significantly (*R* ^2^ = 0.916, *P* < 0.001); (b) number of hospitalizations per year, number of hospitalizations increased more significantly in CD patients (*R* ^2^ = 0.937, *P* < 0.001); (c) proportion of each subtype of IBD changed from 2003 to 2011. Population data of IBD patients and hospitalizations were collected from Renji hospital between Jan, 2003 and Dec, 2011 and from Ruijin hospital between Jan, 2010 and Dec, 2011. Proportion data of IBD patients were collected from Renji hospital between Jan, 2003 and May, 2012 and Ruijin hospital from Aug, 2009 and Jun, 2012. (d) The trend of length of stay.

The number of hospitalized IBD patients (*R* ^2^ = 0.932, *P* < 0.001) and hospitalizations (*R* ^2^ = 0.958, *P* < 0.001) increased significantly from 2003 to 2011 (Figures 1(a) and 1(b)). Interestingly, we found that CD increased much more significantly than UC both on the number of patients (*R* ^2^ = 0.916, *P* < 0.001 for CD; *R* ^2^ = 0.147, *P* = 0.308 for UC), and hospitalizations (*R* ^2^ = 0.937, *P* < 0.001 for CD; *R* ^2^ = 0.376, *P* = 0.079 for UC), which indicated that CD had increased as the majority of IBD subtypes. Consequently, the rates of CD/UC among hospitalizations increased from 0.035 in 2003 to 1.639 in 2012 (Figure 1(c)).

Furthermore, the ratio of male to female (M/F) was 2.04 in patients with CD and 1.24 in patients with UC, respectively. The gender composition of CD (*P* = 0.530) and UC patients (*P* = 0.157) did not show significant changes in the past decade. Due to the changes of medical insurance policy in these ten years, it was demonstrated a significant descending tendency of LOS in patients with CD during the past 10 years (*R* ^2^ = 0.541, *P* = 0.015). We also found the same tendency of LOS in patients with IBDU (*R* ^2^ = 0.722, *P* = 0.009). However, LOS did not significantly alter in patients with UC from 2003 to 2012 (*R* ^2^ = 0.251, *P* = 0.140).

**Figure 2:**
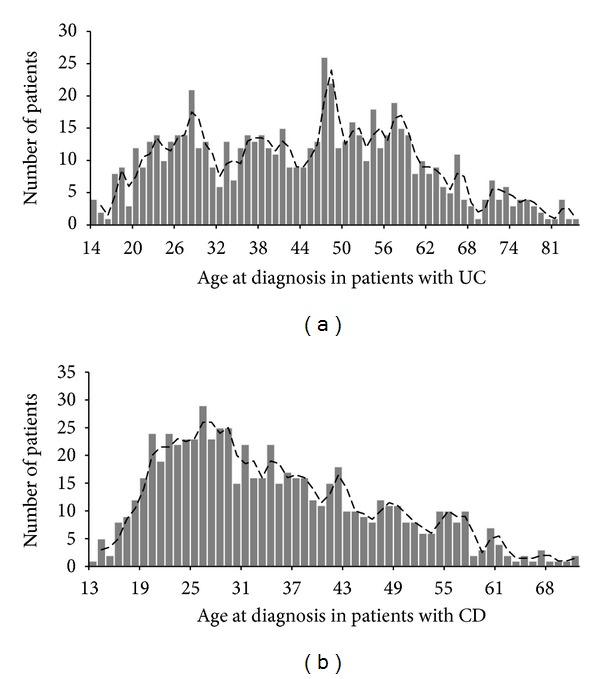
Age distribution of UC (a) and CD patients (b) for their first hospitalization.

The mean age at diagnosis of 668 CD patients was 34.47 yrs (95% CI = 33.51–35.43 yrs). For 665 UC patients, the mean age at diagnosis was 44.10 yrs (95% CI = 42.88–45.31 yrs), and the mean age at diagnosis of IBDU patients was 44.65 yrs (95% CI = 40.44–48.85 yrs). The mean age at diagnosis was significantly younger in CD patients than in UC patients (34.47 yrs versus 44.10 yrs, *P* < 0.0001). Interestingly, we found that the first peak for age at diagnosis in patients with UC was around 28 years old, and the second was around 47 years old (Figure 2(a)). However, the only peak for age at diagnosis in patients with CD was around 26 years old (Figure 2(b)).

### 3.2. Alterations of Hospitalization Costs

**Figure 3:**
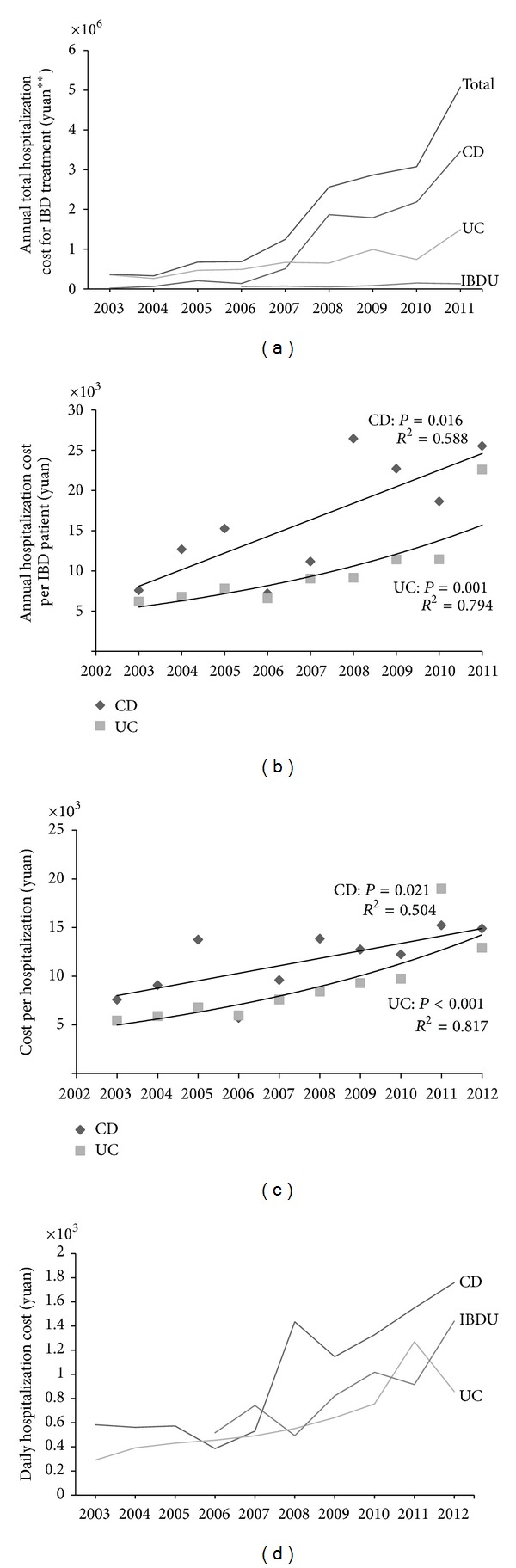
Alterations of hospitalization costs. (a) The variation trend of annual total hospitalization cost for IBD treatment from 2003 to 2011; (b) linear regression line of annual hospitalization cost per CD patient and exponential regression curve of annual hospitalization cost per UC patient from 2003 to 2011; (c) linear regression line of the cost per CD hospitalization and exponential regression curve of the cost per UC hospitalization from 2003 to 2012; (d) daily hospitalization cost.

The annual total hospitalization cost for IBD treatment increased significantly in the past decade (¥ 367268.21 in 2003 versus ¥ 5080726.24 in 2011, *R* ^2^ = 0.956, *P* < 0.001) (Figure 3(a)). It was shown that the annual total hospitalization cost for CD treatment increased more rapidly (¥ 15145.36 in 2003 versus ¥ 3459768.21 in 2011, *R* ^2^ = 0.922, *P* < 0.001) although the annual total hospitalization cost for UC treatment also increased (¥ 352122.85 in 2003 versus ¥ 1490603.49 in 2011, *R* ^2^ = 0.857, *P* < 0.001). However, no significant difference was observed among the annual total hospitalization cost for IBDU treatment in the past decade (*R* ^2^ = 0.646, *P* = 0.054).

We found that the annual hospitalization cost per patient (¥ 7572.68 in 2003 versus ¥ 25504.21 in 2011, *R* ^2^ = 0.588, *P* = 0.016) and the cost per hospitalization (¥ 7572.68 in 2003 versus ¥ 14873.69 in 2012, *R* ^2^ = 0.504, *P* = 0.021) significantly increased in patients with CD. Furthermore, the annual hospitalization cost per patient (¥ 6177.59 in 2003 versus ¥ 22584.90 in 2012, *R* ^2^ = 0.794, *P* = 0.001) and the cost per hospitalization (¥ 5417.27 in 2003 versus ¥ 12910.50 in 2012, *R* ^2^ = 0.817, *P* < 0.001) in patients with UC also increased significantly. We found that the annual hospitalization costs per patient and costs per hospitalization were higher in CD patients than in UC patients (Figures 3(b) and 3(c)). Unreliable trends were excluded for patients with IBDU because of the insufficient sample size.

Moreover, our data showed that the daily cost during hospitalization increased significantly in CD patients (*R* ^2^ = 0.775, *P* = 0.001), UC patients (*R* ^2^ = 0.885, *P* < 0.001), and IBDU patients (*R* ^2^ = 0.750, *P* = 0.012). As shown on the figure, the daily cost during hospitalization in patients with CD is higher compared with UC patients (¥ 1325.63 versus ¥ 681.87, 1.94 folds in CD than UC) or IBDU patients (¥ 1325.63 versus ¥ 856.84, 1.55 folds in CD than IBDU) in these ten years (Figure 3(d)).

### 3.3. Distribution of Hospitalization Costs and Alterations

#### 3.3.1. Medication and Infliximab

**Figure 4:**
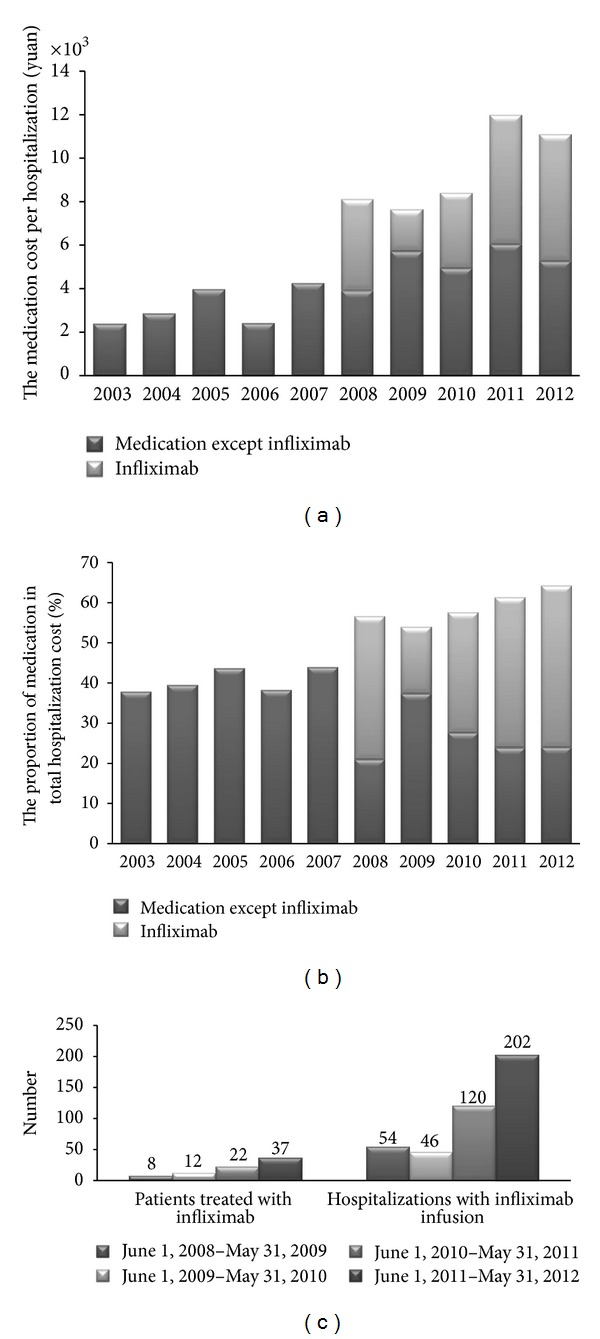
Medication and infliximab costs in IBD. (a) The medication cost per hospitalization; (b) the proportion of medication in total hospitalization cost; (c) the population of patients treated with infliximab and the number of hospitalizations with infliximab infusion.

In the past 10 years, the medication cost per hospitalization was significantly higher in patients with CD than in patients with UC (¥ 10931 versus ¥ 6763, *P* < 0.001) or in patients with IBDU (¥ 10931 versus ¥ 6578, *P* < 0.001). Yet, no significant difference of the medication cost per hospitalization was observed between UC and IBDU patients (*P* = 0.597). As was known in the present study, the medication cost per hospitalization (*R* ^2^ = 0.879, *P* < 0.001) and the proportion of medication in total hospitalization cost (*R* ^2^ = 0.890, *P* < 0.001) significantly increased over this decade, especially in the recent 5 years (Figures 4(a) and 4(b)).

Since we realized that infliximab might be a factor leading to the growing cost (Figures 4(a) and 4(b)), the pattern of infliximab affecting the total hospitalization cost was analyzed. 9.67% of the patients received infliximab treatment from 2008 to 2012, including 11.97% of the CD patients and 2.03% of the UC patients. No patients with IBDU were treated with infliximab. The population of patients treated with infliximab increased significantly (*R* ^2^ = 0.995, *P* = 0.003) and the number of hospitalizations with infliximab infusion only showed an increasing trend (*R* ^2^ = 0.854, *P* = 0.076, Figure 4(c)).

It was found that, in hospitalizations with infliximab infusion, the hospitalization costs were significantly higher than hospitalizations without infliximab infusion during 2008–2012 (¥ 23655.98 versus ¥ 10539.91, *P* < 0.001). Moreover, the present study also found that the daily costs in hospitalizations with infliximab infusion were higher compared with hospitalizations without infliximab infusion (¥ 6003.50 per day versus ¥ 809.20 per day, 7.42 folds in IFX group than the other).

#### 3.3.2. Examination and Endoscopy

**Figure 5:**
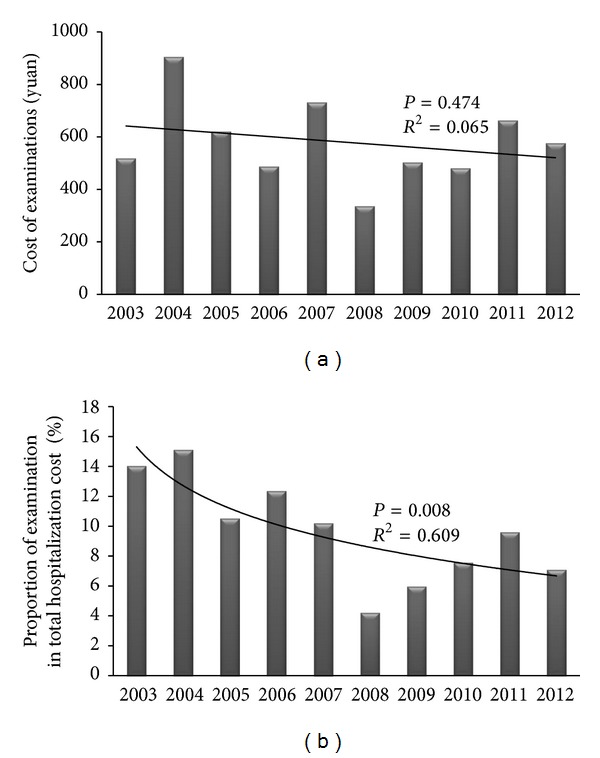
Cost of examinations. (a) Cost of examinations; (b) the proportion of examination in total hospitalization cost.

The linear regression line for cost of examinations was shown in Figure 5(a). We could not find any significant difference in the past 10 years (*R* ^2^ = 0.065, *P* = 0.474). However, it was found that the proportion of examination in total cost significantly decreased in this decade (*R* ^2^ = 0.609, *P* = 0.008, Figure 5(b)), which might be due to the rise of medication cost.

**Figure 6:**
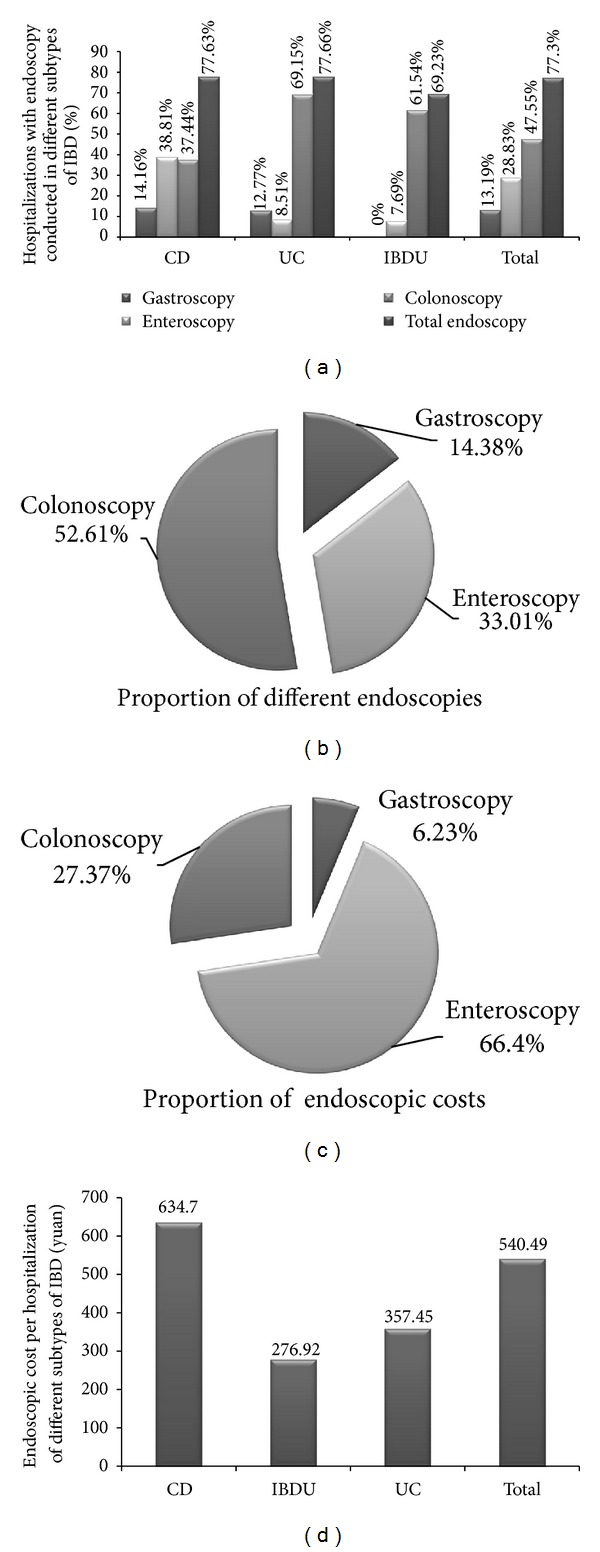
Endoscopies used in IBD determination or evaluation. (a) Percentage of hospitalizations with endoscopy conducted in different subtypes of IBD; (b) the proportion of different endoscopies; (c) the proportion of endoscopic costs; (d) endoscopic costs per hospitalization of different subtypes of IBD.

77.30% inpatients with IBD underwent at least one kind of endoscopic examinations including gastroscopy (13.19%), enteroscopy (28.83%), and colonoscopy (47.55%). Enteroscopy was more frequently used in hospitalizations of patients with CD (38.81%) than hospitalizations of patients with UC (8.51%, *P* < 0.001) or IBDU (7.69%, *P* = 0.050). Only 37.44% of the hospitalizations of CD patients underwent colonoscopy, while it was 69.15% and 61.54% for the hospitalizations of UC patients (*P* < 0.001) and IBDU patients (*P* = 0.083) (Figure 6(a)). Colonoscopy was the most frequently used endoscopic approach (52.61%, Figure 6(b)). However, enteroscopy took up the majority of endoscopic cost (66.40%, Figure 6(c)). In average, it took ¥ 540.49 per hospitalization for endoscopy in patients with IBD, which was accounted for 45.28% of the examination cost but only 2.62% of the hospitalization cost. Moreover, the costs of endoscopy were significantly higher in hospitalizations of CD patients compared with hospitalizations of UC patients (¥ 634.70 versus ¥ 357.45, *P* < 0.001) and IBDU patients (¥ 634.70 versus ¥ 276.92, *P* < 0.001) (Figure 6(d)).

#### 3.3.3. Surgery

**Figure 7:**
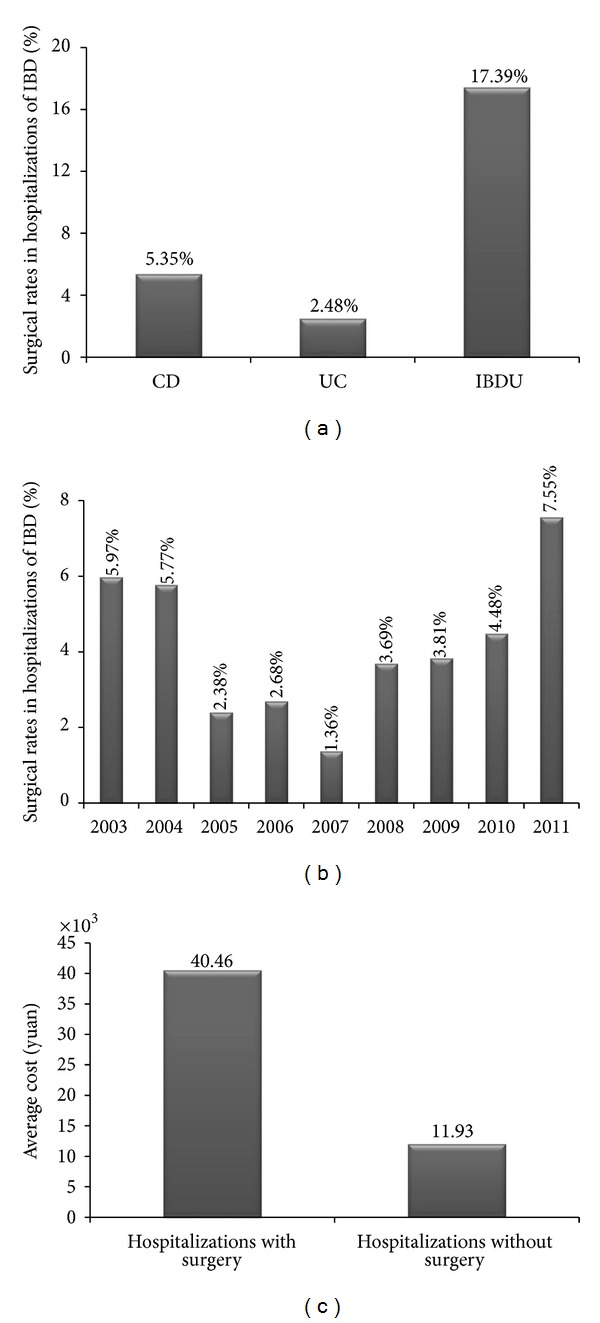
Surgery and costs in IBD. (a) Surgical rates in hospitalizations of IBD; (b) surgical rates changed in the last decade; (c) the costs were significantly higher in hospitalizations with surgery than in hospitalizations without surgery.

A total surgical rate was 4.47% in all hospitalizations with 5.35% in CD, 2.48% in UC, and 17.39% in IBDU. Thus, significant difference of surgical rates could be observed within different subtypes of IBD (*P* < 0.001) (Figure 7(a)). However, there was no significant difference within the annual surgical rates from 2003 to 2011 although we found a growing trend since 2007 (*P* = 0.098, Figure 7(b)). Furthermore, the hospitalization costs were significantly higher in hospitalizations with surgery than in hospitalizations without surgery (¥ 40460 versus ¥ 11933, *P* < 0.001, Figure 7(c)). However, neither the surgical cost per hospitalization (*R* ^2^ = 0.245, *P* = 0.146) nor the proportion of surgery in hospitalization cost (*R* ^2^ = 0.227, *P* = 0.164) significantly changed in the last decade.

### 3.4. Factors Associated with Hospitalization Cost Alterations

To identify the factors associated with hospitalization cost alterations, the ordinal logistic regression model was used (Table 1). We noticed that infliximab infusion contributed significantly to the elevated costs in hospitalizations of IBD (OR = 44380.09, *P* < 0.001). Higher ages, long length of stay, without medical insurance, CD or IBDU, worse prognosis, surgery, and endoscopy were also factors associated with higher hospitalization costs in patients with IBD (all *P* < 0.05).

## 4. Discussion

### 4.1. Increase of Hospitalizations and Different Peaks for Age at Diagnosis

The incidence and prevalence of IBD are increasing in different regions around the world. It has been reported that the incidence and prevalence of IBD have been stable or slightly increased in Northern and Western Europe as well as North America in recent years [4, 7]. However, a significant rise had been found in some areas of Southern and Western Europe as well as Asia these years, with low incidence and prevalence in the past [18, 19]. The gap between areas with high and low incidence rates is diminishing [5].

According to the data in central China, the prevalence of CD and UC increased between 1990 and 2003 [20]. Our study also partly reveals the fact that the population of IBD had been continuously increasing in recent years, which may be due to development in medical technique and equipment and deeper understanding of the clinical characteristics and pathogenesis of IBD. However, the rapid increase is unlikely to be fully accounted by the improvement of diagnostic method and better awareness of IBD. The significant increase in China seems to parallel the improvement of life quality and the rise in GDP, leading us to consider more about environmental factors to pathogenesis of IBD [8]. It was reported that fried food intake and stress in study or work were risk factors for IBD, which indicates that changes of life style also contributed to the increased incidence [21]. 

In the present study, we found that the number of hospitalized CD patients and CD hospitalizations increased much more rapidly compared with patients with UC or IBDU. The widely used enteroscopy, experienced endoscopists, and advent of capsule endoscopy in recent years made the detection rate of CD patients increase rapidly, which could be crucial factors for faster increase than UC patients [22, 23]. More interestingly, it has been reported that the pattern of prevalence in the Chinese population was different from the pattern in Western countries, that is, no second peak age of onset in Chinese patients with CD or UC [24]. However, our study found that the first peak for age at diagnosis in patients with UC was around 28 years old and the second was around 47 years old. To our knowledge, it is the first time that double peaks for age at diagnosis were found in Chinese patients with UC, which might show the alteration of onset in this decade.

### 4.2. Growing Hospitalization Cost and Associated Factors

We found that the annual total hospitalization cost for IBD treatment increased significantly in the last ten years. It was also noticed that the annual total hospitalization cost for CD treatment, annual hospitalization cost per CD patient, and cost per CD hospitalization increased faster than UC. The faster growth of costs in CD patients and treatment was driven by different characteristics among subtypes of IBD. First, extraintestinal manifestations and complications were more common in CD patients, which make the treatment of CD difficult [25]. Second, considerable progression of enteroscopy, more experienced endoscopists, and advents of other endoscopic and radiographic techniques in recent years made more thorough evaluation of the small intestine possible, which remarkably elevated the detection rate of Crohn's disease [23, 24]. Third, the biological agent, infliximab, was mostly used for treating patients with CD, but rarely used in patients with UC in China to date.

Infliximab was mostly used in a selected minority of IBD patients who did not adequately respond to conventional therapies, especially in CD patients. In many countries, it was reported that the interest in the economics of managing IBD has heightened with the advent of costly biological agents, which gradually became the main driver for healthcare cost [10, 14]. However, the shift in cost profile was not so obvious in European population, according to the results from a large cohort study in Europe and a study in UK, respectively [11, 12]. In the present study, from 2008 to 2012, 9.67% of the patients received infliximab treatment, including 11.97% of CD patients and 2.03% of UC patients, which was much higher than the proportion reported in Canada (1.5%) but lower than the proportion reported in Netherlands (23% in CD patients; 4% in UC patients) [9, 14]. Interestingly, this proportion was still rising according to our study. We also found that, in recent years, infliximab became a major predictor of higher medical cost, representing a shift in medical costs of IBD patients in China, in line with the result in The Netherlands and US as reported [10, 14].

With the development of evidence based medicine and understanding of mucosal healing, endoscopic presentation became more critical in diagnosis, evaluation, and surveillance of cancerous lesions in patients with IBD [26, 27]. According to our result, colonoscopy was the most frequently used endoscopic examination, which might be partly attributed to the common lesion in colon for IBD patients. The costs of endoscopy were significantly higher in hospitalizations of CD patients than UC or IBDU patients, mainly due to the particular involvement in small intestine for CD patients. Although there was a majority of CD patients undergoing colonoscopies, the discontinuous lesions occurring in the small intestine notably increased the demand of enteroscopies (much more expensive than colonoscopies) for patients with CD, and as a result, enteroscopies were more frequently used in CD patients than in UC or IBDU patients. Furthermore, as mucosal healing becoming a critical objective sign of remission and gaining more acceptance as a measure of disease activity in recent years, regular use of endoscopy was required for assessment of mucosal healing [28].

It has been reported that 12% of CD patients underwent intestinal resections and 6% of UC patients underwent proctocolectomy [29]. Another study from South Asia found that 14.1% of the IBD patients underwent surgery [30]. In the present study, our result indicated relatively lower surgery rates in patients with IBD (5.35% in CD, 2.48% in UC, and 17.39% in IBDU, resp.). Our result revealed that CD patients had relatively higher risk for surgery than UC patients, partly because the patients with CD needed small surgeries to induce transient remission or solve complications as perianal abscesses or anal fistula. However, we found the surgery rates of IBD patients did not change significantly in the past years, maintaining a low level for several years, unlike the general decreasing trend in Europe and North America [31, 32]. It was reported that more than half of the patients would encounter endoscopic relapse and part of them would ultimately clinically relapse after surgery, which required more surgical interventions to manage the obstruction or perforation [33]. Furthermore, postoperative recurrence seemed difficult to accept for many IBD patients, which may be the primary reason for low surgery rate in China. In fact, the introduction of infliximab might partly take the place of surgery, which might be the primary reason for low level on surgery rate rather than increasing [10, 14].

### 4.3. Insurance Principles Control the Factors Associated with Growing Cost of Hospitalization

Medical insurance and the LOS are two factors associated with insurance principles. The growth of healthcare cost has increased rapidly in many countries [34, 35]. Health care reforms were enacted in some countries to bend the rising health care cost curve [36, 37]. In Canada, several features of its program helped constrain cost [38]. Canadian hospitals received prospectively determined global operating budgets, in which capital costs were not involved but distributed separately, eliminating incentives to overprovide lucrative services. In addition, Canada's provincial plans used their intensive purchasing power to limit the prices of drug and device, effectively constraining the healthcare cost globally.

In China, the cost of health care increased dramatically over the past decades [39]. With increasing costs, accessing health care became more difficult for those who could not pay and medically induced poverty emerged [40]. Thus, greater attention was paid to health reform. In the present study, it was suggested by our result that medical insurance provided by the Chinese government could control the reasonable expenses efficiently in management of IBD. Similar to many western countries, in recognition that strong incentives for overutilization of unnecessary services and medicines were provided by user fee systems, more efforts were paid to improve payment methods. The essential medicines list (EML) had been issued which could ensure the availability of a group of basic medicines and offered a relatively higher reimbursement rate for them compared with medicines not on the list [41]. Adopting evidence-based approaches to help select medicines explicitly and reasonably in EML and zero profit mark-up policy for essential medicines would also help reduce cost [41].

Reduction of LOS was another emphasis of hospitalization cost control. Our result suggested that shortening LOS helped improve the efficacy of both diagnosis and treatment, effectively controlling the hospitalization cost to some extent. However, the daily cost during hospitalization increased in the last decade, especially from 2008 to 2012, which is mainly attributed to the increasing use of biological agent. The daily cost during hospitalization became much higher in CD patients than UC patients, which may also be largely due to more infliximab infusions in CD patients.

In conclusion, we found a significant rise in hospitalized IBD patients, frequency of hospitalizations, total hospitalization cost for IBD treatment, annual hospitalization cost per patient, cost per hospitalization, and daily cost during hospitalization over the last decade. The hospitalized patients, frequency of hospitalizations, and hospitalization cost increased more rapidly in CD than in UC and IBDU. Double peaks for age at diagnosis in Chinese patients with UC were found for the first time. Infliximab had already become the biggest driver for high medical cost, shifting the pattern of costs in IBD patients in China. Endoscopic costs in hospitalizations of CD patients were significantly higher than those of UC and IBDU. A relatively lower surgery rate was found. Other than infliximab infusion, higher ages, long length of stay, being without medical insurance, CD or IBDU, worse prognosis, surgery, and endoscopy were also factors associated with higher hospitalization costs in patients with IBD. The medical insurance efficiently controlled the reasonable expenses in management of IBD by providing essential medicines list, adopting evidence-based approaches, and shortening LOS.

## Figures and Tables

**Table 1:** Factors associated with hospitalization cost alterations.

Characteristics of hospitalization	*N*	Percentage	OR (95% CI )	*P* value
Age	1654	100%	**1.01 **(**1.00**–**1.02**)	**0.009**
LOS*	1654	100%	**1.29 **(**1.26**–**1.31**)	**<0.001**
Gender				
Male	1027	62.09%	1.23 (0.99–1.54)	0.063
Female	627	37.91%	1.00	
Insurance status				
Without medical insurance	778	47.04%	**1.31 **(**1.05**–**1.64**)	**0.017**
Medical insurance	876	52.96%	1.00	
Disease subtypes				
CD	841	50.85%	**3.55 **(**2.79**–**4.52**)	**<0.001**
UC	767	46.37%	1.00	
IBDU	46	2.78%	**4.30 **(**2.32**–**7.97**)	**<0.001**
Prognosis				
Dead or not improved	21	1.27%	**6.78 **(**2.72**–**16.90**)	**<0.001**
Cured or improved	1633	98.73%	1.00	
Urgency on admission				
Critical or urgent	591	35.73%	1.18 (0.94–1.47)	0.151
Normal	1063	64.27%	1.00	
Surgical intervention				
Yes	72	4.35%	**3.16 **(**1.71**–**5.82**)	**<0.001**
No	1582	95.65%	1.00	
Biological agent (infliximab)				
Yes	264	15.96%	**44380.09 **(**15936.73**–**123588.19**)	**<0.001**
No	1390	84.04%	1.00	
Endoscopy**				
Yes	253	44.46%	**2.44 (1.50**–**3.96)**	**<0.001**
No	316	55.54%	1.00	

*Length of stay.

**Data from Apr, 2010 to Mar, 2012 in Renji hospital.

Bold values were with significance (*P* < 0.05).
